# Integration of arts and humanities in medicine to develop well-rounded physicians: the roles of health sciences librarians

**DOI:** 10.5195/jmla.2022.1368

**Published:** 2022-04-01

**Authors:** Misa Mi, Lin Wu, Yingting Zhang, Wendy Wu

**Affiliations:** 1 mi@oakland.edu, Interim Director, Medical Library; Professor, Department of Foundational Medical Studies; Oakland University William Beaumont School of Medicine Medical Library, Rochester, MI; 2 lwu5@uthsc.edu, Assistant Director/Associate Professor, Research & Learning Services, Health Sciences Library, University of Tennessee Health Science Center, Memphis, TN; 3 yzhang@libraries.rutgers.edu, Research Services Librarian, RWJ Library of the Health Sciences; Adjunct Assistant Professor, Department of Medicine; Rutgers, The State University of New Jersey, New Brunswick, NJ; 4 wendy.wu@wayne.edu, Librarian IV, Shiffman Medical Library, Wayne State University; Detroit, MI

**Keywords:** arts, humanities, health sciences library, undergraduate medical education, medical education, health sciences librarians

## Abstract

Over the past ten years, there has been a growing interest in integrating arts and humanities in medicine to increase learners' empathy and resilience; improve personal well-being, communication, and observational skills; enhance self-reflection; and promote professionalism. These desired skills and qualities are becoming increasingly important for the physicians of tomorrow. Parallel to curricular interventions of integrating arts and humanities to medical education, there has been an increasing research interest in investigating the impact of such interventions on medical students with respect to improving and sustaining students' empathy as they progress in their medical education and develop their professional identity. Research has yielded interesting findings on the types and effect of the interventions in the medical curriculum. The Association of the American Medical Colleges (AAMC), recognizing the unique and unrealized role of arts and humanities in preparing and equipping physicians for twenty-first-century challenges, proposed seven recommendations for advancing arts and humanities integration into medical education to improve the education, practice, and well-being of physicians and physician learners across the spectrum of medical education. Institutional initiatives of arts and humanities integration in the medical curriculum in response to the AAMC's recommendations afford health sciences librarians expansive opportunities and a new landscape of playing an important role in these initiatives. With their diverse educational background in arts, humanities, social sciences, and many other disciplines and fields, health sciences librarians are poised for meaningful contributions to their institutional goals in developing a humanistic, compassionate workforce of future physicians.

## INTRODUCTION

Human beings use the arts as the vehicles to express the meanings of their lives [1]. Medical humanities can promote a depth of human and humane understanding, knowledge, and experience [2]. The humanities force reflective thinking and contemplation skills that are essential to thoughtful decision-making and to personal wellness. Beyond that, in Danielle Ofri's opinion, the humanities add “a dose of joy and beauty” to an arduous training process that is notoriously barren in these areas. Well integrated into the training, the humanities can be the kernel of transforming medical knowledge into clinical wisdom [3]. John Gillies states that “arts and humanities approaches are intrinsic to society's understanding of medicine” [4]. In treating and caring for sick and often distressed patients, physicians repeatedly confront fundamental questions about the meaning and sanctity of life and the problem of suffering [5], and they grapple with uncertainty and ambiguity in clinical decision-making. Gillie Bolton believes that “a sound grounding in the arts and humanities can enable an effectively critical, humane, and ethical response” [5].

Evidence-based medicine (a systematic approach to medicine) is to use the current best available evidence from patient-focused clinical research to inform clinical decision-making in the care of individual patients. However, a clinician's clinical judgment, expertise, experience, and the patient's values and preferences are equally important in the process of clinical decision-making [6]. Furthermore, in patient encounters, clinicians bring their own perspectives or personal experiences; individual patients bring their afflicted bodies or troubled minds. Howard Brody expressed his sentiment by saying, “Often more than the body or mind is broken: patients' understanding of themselves and the certainty of their lives is often also disrupted and disturbed” [7]. Apart from that, patients' hearts could be troubled and shattered. The use of clinical evidence is for treating and curing the damaged and afflicted body in the physical sense, but the minds and emotions associated with the brokenness have to be examined and attended to through incorporating the lens of the humanistic view with compassion and empathy. Attention to the patient's role in both evidence-based medicine and humanistic medicine could help create a holism in medicine where the clinician and the patient can “co-construct a curative and healing narrative that involves medical and healthcare interventions, and mental and emotional understandings, which can help the patient constructively rebuild their life, or prepare for death” [7].

How can medical educators use arts and humanities (AH) as a means or vehicle to instill a humanistic view of medicine in learners across the spectrum of medical education? How can medical educators help mitigate learners' stress and burnout in face of the many challenges in completing medical school?

The Liaison Committee on Medical Education establishes standards for accreditation of medical education programs leading to the MD degree in the United States and Canada. Among all the standards, Standard 10 is related to medical student selection, assignment, and progress. It states:

Through its requirements for admission, a medical school encourages potential applicants to the medical education program to acquire a broad undergraduate education that includes the study of the humanities, natural sciences, and social sciences, and confines its specific premedical course requirements to those deemed essential preparation for successful completion of its medical curriculum [8].

The Association of American Medical Colleges (AAMC) has instigated efforts in strengthening the medical school applicant selection by developing fifteen core competencies for entering students. These core competencies are used to form selection and admission criteria for medical school applicants. To be successful, applicants must demonstrate certain skills, knowledge, and abilities in areas such as social skills, cultural competence, resilience and adaptability, oral and written communication, and human behavior [9]. AAMC also took its stand on the role of arts and humanities in undergraduate medical education by stating, “By integrating arts and humanities throughout medical education, trainees and physicians can learn to be better observers and interpreters; and build empathy, communication and teamwork skills, and more” [10]. Clearly, AAMC recognized the unique and unrealized role of AH in preparing and equipping physicians for twenty-first-century challenges in contributing to optimal health care outcomes for patients and communities. The organization proposed seven recommendations for advancing AH integration into medical education to improve the education, practice, and well-being of physicians and physician learners across the spectrum of medical education [10]. The recommendations focus on: (1) practicing medicine as an art as well as a science to develop a deep understanding of the human conditions in trainees and physicans; (2) creating more effective AH integrated models for competency-based teaching and learning in medicine; (3) enhancing the research and evaluation of AH integrated courses and programs; (4) designing AH integrated approaches to enhancing trainee and physican well-being; (5) increasing interdisciplinary or multidisciplinary collaboration among scholars and learners and patients; (6) providing professional development offerings on designing and using AH integrated models in medical education; and (7) investigating the effectiveness of AH integrated educational practice and recognizing the value of scholarship in such research endeavors in academic promotion and tenure process [10]. Clearly, these seven recommendations point to strong implications for medical educators and researchers as well as health sciences libraries.

Over the past decade, there has been a growing interest in integrating AH into health profession educational programs to increase learners' empathy and resilience; improve personal well-being, communication, and observational skills; enhance self-reflection; and promote professionalism. These skills and qualities are becoming increasingly important for the physicians of tomorrow. According to the AAMC Curriculum Inventory, during the year of 2015–2016, humanities education was required in 119 medical schools. While there has been a growth in teaching humanities in the medical curriculum, there has been a lack for a “deep, sustained, foundational, across-the-board incorporation into all medical schools” [11].

Parallel to AH interventions in the medical curriculum, there has been an increasing research interest in investigating the impact of AH interventions on medical students with respect to improving and sustaining their empathy, and developing their communication, observational, and other important skills as they progress in their education and form their professional identity. Previous research has yielded promising findings on the efficacy of AH interventions in the medical curriculum.

We conducted a comprehensive review of the literature to find out how AH was integrated in medical education and what impact the integration had on students during the critical years of their identity formation in medical school. The literature review reveals that different types of AH intervention were developed and implemented in the medical curriculum. Artwork was most used in interventions, followed by film, theater or drama, humor, patients' stories, and comic strips. The artwork used in the interventions came from various sources, including a gallery or museum [12–14], patients with chronic mental illness [15], medical students participating in research studies [16–18], and patients in collaboration with students [19]. The artwork was used as a vehicle to present a wide array of learning experiences to examine the impact of artistic creativity on life, students' understanding of palliative care [12], observational skills [13, 14, 20], level of empathy [17, 20, 21], and communications skills with an attitude toward older adults [19] or chronically ill older patients [18].

Another way of embedding AH into the curriculum was using movies to address topics of professionalism, mental illness, empathy, patient encounters, bias toward patients with mental illness, and psychological stress. Examples of these interventions include students watching a movie with still and moving images with music but stripped of dialogue [22]; students involved in movie screening, critiquing, analyzing, and comparing and contrasting the representation of mental illness, psychiatry, or psychiatrists [23]; students watching a movie that ended with interviews with former medical students and body donors in the movie [24]; and students watching video clips from movies and discussing patient encounters [25].

Theater or drama techniques were applied in interventions to teach students about breaking bad news or delivering death notification [26, 27], to develop positive professional qualities and interpersonal skills [28], and to sustain or enhance empathy [29]. Other AH interventions attempted to impart to students fundamental skills for a humanistic physician by using humor [30], patients' stories [31], or comic strips [32].

AH interventions were implemented throughout medical education, but more were found in the curricula of the first year or clinical years. Reported outcomes were primarily in seven categories: (1) enhanced compassion or empathy, (2) improved communication skills, (3) enhanced professionalism or professional identity, (4) improved observational skills, (5) increased humanistic view, (6) reduced burnout/stress, and (7) enhanced physician-patient relations. The outcome data seem promising and thus point to the importance and value of AH integration and resulted outcomes in medical and other health profession educational programs in developing a future workforce of humanistic health care providers.

## ROLE OF HEALTH SCIENCES LIBRARIES AND LIBRARIANS

Many health sciences libraries and librarians have been successful in exerting influence on many institution-wide initiatives and programs in various areas, such as ongoing support for clinical or biomedical research, teaching evidence-based medicine, forging partnerships with faculty in teaching students, and educating or assisting researchers in conducting systematic reviews. Institutional initiatives in AH integration in medical education in response to the AAMC's recommendations afford health sciences librarians a new landscape of opportunities for participating in these initiatives. With their rich and diverse backgrounds, expertise in information management, and organizational skills, coupled with their ability to reach out to communities and work with multidisciplinary teams, health sciences librarians are well positioned to contribute to their institutional goals in developing humanistic and compassionate physicians. It is time for librarians to leverage their multifaceted role and skill sets to lead or participate in initiatives and efforts in integrating arts and humanities into the teaching and learning practices at their home institutions.

The literature review gave us the lay of the land and shed light on multiple approaches to AH integration and its impact on medical students. The growing interest in and attention to the integration in medicine, investigating its impact fueled by the AAMC initiative for the FRAHME (the Fundamental Role of the Arts and Humanities in Medical Education), and the seven recommendations for integrative arts and humanities curricula implicate great potential for health sciences librarians in innovating and expanding their role to exert an impact in health profession educational programs. What follows are a few recommendations for health sciences librarians who desire to expand their roles, innovate their library services and programs, and build communities and forge multidisciplinary collaboration, interaction, and partnerships with different groups of constituents at their home institution.

1. *Community engagement*. Health sciences librarians have long been active in reaching out to the community and serving as a conduit bridging the distance between the academia and the wider community. Librarians can help reinforce the community engagement of their home institution by planning and organizing activities like art shows, photo galleries, movie nights, book clubs, or other events to connect students, faculty, and staff with community members to enrich students' campus life and learning experience.
2. *Curriculum development*. Health sciences librarians have diverse and rich backgrounds in different disciplines and fields, and their talents and expertise could be tapped in developing outreach or educational programs. Health sciences librarians with educational backgrounds in arts, humanities, and social sciences could participate in or lead efforts in developing and teaching in sessions, elective courses, or programs that incorporate this disciplinary knowledge in the forms of creative or narrative writing, movie screening and discussion, fiction or nonfiction book discussion, poetry writing and reciting, art or music appreciation, etc.
3. *Library's physical space*. A health sciences library is more than a physical building of collections of resources or for study space; it can serve as the centerpiece in the landscape of health or medical humanities integration. Libraries strive to serve as a cultural hub for community engagement as well as a center for student academic life. The library's physical and virtual space could be reorganized or restructured to accommodate diverse needs for creating and promoting AH-embedded learning events and activities for students, faculty, staff, and the wider community. The library physical space—available and desirable as an art gallery and for photo exhibits, book clubs, or music performance—could enhance participants' experiences and meet their needs for in-person interaction and socialization.
4. *Library's virtual space*. The library's website has increasingly become students' and faculty's primary access point to many library resources, services, and program offerings. The virtual space could be redesigned to aid in promoting AH-related events and activities and facilitating quick and easy access to and efficient and effective use of resources across disciplines. These resources can be made available and accessible through a searchable depository of digitized artifacts, online photos, literary works, movies, music, poetry, and other art forms to support efforts in AH initiatives or interventions in health profession education programs.
5. *Library programs*. Health sciences libraries can organize and host programs or events such as faculty and student art exhibits and contests, talent shows, movie nights, book clubs, and poetry readings to foster faculty's and students' exploration of and discussion on the human dimensions of culture, illness, health, and health care. These events and programs lend themselves well to forging librarians' collaboration and partnership with faculty across disciplines to develop curricular or extracurricular learning experiences.

In this digital age, with increasing online learning and remote access to teaching and learning resources, health sciences libraries are propelled to take innovative and creative approaches to reaching out to their constituents and wider communities. The AH integration affords health sciences libraries great opportunities for expanding their endeavors to advance health humanities scholarship, education, and practices through multidisciplinary methods focusing on the intersection of arts, humanities, culture, health, illness, and health care.

## CONCLUSIONS

Health sciences librarians have been on the front line of teaching evidence-based medicine and facilitating use of and access to evidence-based resources. The scientific research evidence from evidence-based resources informs clinicians' decision-making on the care for patients, treating and curing the physical suffering. However, the distressed inner minds and turbulent emotions associated with the shattered, troubled hearts resulting from the damaged body may need to be attended with the lens of the humanistic, holistic view with compassion and empathy. The AH integration into health profession educational programs can well serve as an intervention to instill the humanistic, holistic view in students who will be entrusted to care for patients with compassion and empathy. The increased interest in integrating AH in medicine to develop well-rounded health care providers against the backdrop of the current pandemic provides a fertile ground for health sciences librarians to be creative and innovative in practicing health sciences librarianship. Librarians' involvement in AH interventions would add to their arsenal of skill sets and expertise. These skills would be indispensable to strategic goals of molding and transforming health profession students into future health care providers who contribute to optimal patient care and outcomes. Librarians' involvement in the AH integration may inject a dose of joy and beauty into the learners' academic lives and librarians' professional lives alike.
